# Comparison of the Quality of Chronic Disease Management Between Adults With and Without Dementia

**DOI:** 10.1001/jamanetworkopen.2021.9622

**Published:** 2021-05-13

**Authors:** Hiroshi Gotanda, Teryl Nuckols, Kanon Mori, Yusuke Tsugawa

**Affiliations:** 1 Division of General Internal Medicine, Cedars-Sinai Medical Center, Los Angeles, California; 2 RAND Corporation, Santa Monica, California; 3 Department of Health Policy and Management, UCLA (University of California, Los Angeles), Fielding School of Public Health; 4 UCLA Center for Health Policy Research, UCLA Fielding School of Public Health; 5 Division of General Internal Medicine and Health Services Research, David Geffen School of Medicine at UCLA, Los Angeles

## Abstract

**Importance:**

Adults with dementia may experience poorer quality of chronic disease management because of the difficulty in reporting symptoms and engaging in shared decision-making associated with cognitive impairment.

**Objective:**

To compare the quality of chronic disease management received by adults with and without dementia.

**Design, Setting, and Participants:**

For this cross-sectional study, nationally representative data from noninstitutionalized patients 65 years or older were obtained from the 2002-2015 Medical Expenditure Panel Survey. The control group comprised adults of similarly limited life expectancy without dementia comprised . Data analysis was performed in June 2020.

**Exposures:**

Dementia diagnosis.

**Main Outcomes and Measures:**

Quality of chronic disease management based on 14 individual quality indicators. The association between dementia status and the quality of chronic disease management (3 composite categories: preventive care, diabetes care, and medication treatment) was examined using multivariable linear regression models. Survey weights, sampling strata, and primary sampling unit variables were used to produce national estimates adjusted for nonresponse.

**Results:**

This study included 2506 adults (mean [SD] age, 81.4 [4.7] years; 1259 [49.3%] female; 1243 [50.7%] male), of whom 1335 (53.3%) had a diagnosis of dementia and 1171 (46.7%) did not have a diagnosis of dementia. After adjusting for potential confounders, adults with dementia received lower-quality preventive care compared with adults of similar life expectancy without dementia (adjusted absolute difference [aAD], −6.1 percentage points [pp]; 95% CI, −9.7 to −2.5 pp; *P* = .001). We found no evidence that the quality of care differed in diabetes care (aAD, 1.7 pp; 95% CI, −4.5 to 7.9 pp; *P* = .59) and medication treatment (aAD, 1.0 pp; 95% CI, −5.0 to 7.0 pp; *P* = .75).

**Conclusions and Relevance:**

In this cross-sectional study, the quality of chronic disease management for adults with dementia was not substantially different from that for those without dementia despite potential barriers. Future studies are warranted to gain a better understanding of the underlying mechanism of these findings for preventive care.

## Key Points

**Question:**

Does the quality of chronic disease management provided to adults with dementia differ from the care provided to adults without dementia?

**Findings:**

In this cross-sectional study using national survey data on 2506 adults 65 years or older, participants with dementia received slightly poorer quality of preventive care compared with those without dementia, but both groups received similar diabetes care and medication treatment.

**Meaning:**

In this study, the quality of chronic disease management for adults with dementia was not substantially different from that provided to those without dementia despite potential barriers.

## Teaser

This cross-sectional study compares the quality of diabetes care and medication management provided to adults with dementia with that provided to adults without dementia who had similar limited life expectancy.

## Introduction

Little is known about the quality of chronic disease management provided to adults with dementia in the US. Evidence indicates that adults with dementia are likely to have more comorbid chronic diseases (eg, diabetes, heart failure) than those without dementia, and these comorbidities are associated with a substantial burden among adults with dementia and their caregivers.^1,2,3,4^ Suboptimal control of chronic conditions is associated with a high burden of symptoms and hospitalizations that may substantially disrupt cognitive and physical function.^5^ Identifying gaps in the quality of chronic disease management provided to adults with dementia at the national level is critically important to the design and implementation of interventions to optimize care for this increasing population.

Dementia presents unique challenges to the quality of chronic disease management. Memory deficits and impaired executive function may be associated with limited ability to report symptoms, missed appointments, difficulty in shared decision-making processes, and failure to follow treatment recommendations.^6^ Behaviors associated with dementia, such as aggression and impulsivity, add another layer of complexity to the provision of high-quality chronic disease management. Clinicians should gather collateral information, engage in coordination of care, and make complicated decisions within time constraints. Thus, adults with dementia may be receiving a poorer quality of chronic disease management than those without dementia. However, existing studies^7,8,9,10,11,12,13^ are limited to those conducted outside the US, those based on data obtained from limited regions in the US,^14,15^ or those restricted to nursing home residents^16,17,18^; therefore, the association of dementia status with the quality of chronic disease management among community-dwelling individuals in the US at the national level is largely unknown.

To address this knowledge gap, we compared the quality of chronic disease management for multiple chronic conditions between adults with dementia and those without dementia who had similar life expectancy using a nationally representative sample of older adults living in the community. Care recommendations for older adults in the general population can be irrelevant or even harmful for adults with dementia because of their limited life expectancy and greater risk for adverse events^19^; therefore, we adapted a set of recommended care processes that were previously determined by a panel of experts to be associated with a net benefit for all adults, including those with dementia.^20^ We hypothesized that adults with dementia receive a poorer quality of chronic disease management compared with those without dementia given the potential barriers to providing recommended care.

## Methods

### Data Source and Population

This cross-sectional study used data from the 2002-2015 Medical Expenditure Panel Survey (MEPS), a nationally representative annual survey of the civilian noninstitutionalized population in the US by the US Agency for Healthcare Research and Quality.^21^ The MEPS has an overlapping panel design in which each year a new panel was drawn from households responding to the previous year’s National Health Interview Survey. Participants completed 5 rounds of in-person interviews, a self-administered questionnaire, and the Diabetes Case Survey (if applicable) over 2 years. Collected data included self-reported demographic characteristics, health status, health conditions, and medications. The mean overall response rate was 57.1%.^22^ Our study included data from unique participants 65 years or older who completed the MEPS. Because of the overlapping design of MEPS, the same individual may have appeared in data from 2 consecutive annual files. To avoid the inclusion of data from the same participant, we used the latest data available for a given participant. We excluded 307 observations with missing covariates (eFigure in the Supplement). This study was deemed to be exempt from review by the Cedars-Sinai institutional review board and did not require informed patient consent because all data were publicly available and deidentified. This study followed the Strengthening the Reporting of Observational Studies in Epidemiology (STROBE) reporting guideline.^23^

### Identification of Dementia Status

Dementia status was defined using the *International Classification of Diseases, Ninth Revision, Clinical Modification* (*ICD-9-CM*) diagnosis codes 290.XX, 294.XX, 331.XX, or 797 (MEPS reports 3-digit *ICD-9-CM* codes only), an approach used in previous studies.^24,25^ The medical conditions reported by each MEPS respondent were recorded by the interviewer as verbatim text, and the text strings were coded by professional coders using *ICD-9-CM* codes.^21^

### Identification of the Control Group

Because the median survival time for an individual with dementia is 3 to 10 years from the age at onset of dementia, the control group was defined as adults without a dementia diagnosis who had an estimated 5-year mortality risk of 50% or greater.^26,27^ We used these adults as a comparison group (as opposed to older adults without a dementia diagnosis from the general population) to account for the possibility that clinicians sometimes withhold or withdraw recommended care based on perceived illness.^28^ To estimate a 5-year mortality risk, we adapted a 5-year mortality index using age, sex, body mass index, self-reported general health status, diagnoses (emphysema and/or chronic bronchitis, cancer, and diabetes), basic activities of daily living, instrumental activities of daily living, smoking status, and recent hospitalizations that was developed with the participants of the National Health Interview Survey.^28,29^ To adapt this 5-year mortality index for use with the MEPS data, we mapped MEPS questions to the National Health Interview Survey questions used to compute the mortality index. Although these set of questions are similar, 1 discrepancy is the lack of the “former smoker” option in a question about smoking status in MEPS data (eTable 1 in the Supplement).

### Measurement of the Quality of Chronic Disease Management

We sought quality indicators (QIs)—standardized, evidence-based metrics of health care quality^30^—applicable to adults with dementia who had limited life expectancy and were potentially at risk of adverse health events. We used a framework developed in the Assessing Care of Vulnerable Elders–3 project,^20^ in which a group of clinical experts determined the time horizon of benefits and burden of care for each QI. The time horizon to benefit was classified as short (<6 months), intermediate (6-24 months), or long (≥24 months) because achievement of benefit associated with care may take a substantial amount of time. Similarly, the burden of care was classified as light, moderate, or heavy according to the association of the medical procedure with the preferences of the patient and caregiver. Based on expert opinions, we identified a list of QIs with a short or intermediate time horizon to benefit and low or moderate burden of care that would be associated with a net benefit even for adults with dementia (eTable 2 in the Supplement).

Among the QIs identified using the framework of the Assessing Care of Vulnerable Elders–3 project, we selected 14 QIs that could be evaluated with the MEPS data based on findings from previous studies (Table 1).^31,32^ For each of the 14 QIs, we assessed whether a participant was eligible (eg, had a diagnosis of heart failure) and evaluated whether the participant received a particular process of care to fulfill the QI (eg, β-blocker prescription). We then grouped these 14 QIs into 3 composites (preventive care, diabetes care, and medication treatment) and calculated 3 composite scores by dividing the number of processes of care delivered by the number of processes of care for which a participant was eligible in each composite, an approach used in previous studies.^31,32^ Composite scores provide a useful summary of the quality of chronic disease management; thus, we considered 3 composite scores as the primary outcomes.

**Table 1.** Definitions of Quality Indicators

Quality indicator^a^	Data source^b^	Care to be provided to fulfill an indicator	Eligibility for an indicator
Preventive care			
Influenza vaccine	Self-report	Influenza vaccine within 1 y	Age ≥50 y
Smoking cessation counseling	Self-report	Smoking cessation counseling within 1 y	Smoking
Dental care	Self-report	Dental visit within 1 y	All
Diabetes care			
HbA_1c_ measurement	Self-report	HbA_1c_ measurement at least twice yearly	Diabetes
Foot examination	Self-report	Foot examination within 1 y	Diabetes
Eye examination	Self-report	Eye examination within 1 y	Diabetes
Medication treatment			
Anticoagulation for atrial fibrillation	Verified self-report	Anticoagulation prescription within 1 y	Atrial fibrillation
ACE-I or ARB for heart failure	Verified self-report	ACE-I or ARB prescription within 1 y	Heart failure
β-Blocker for heart failure	Verified self-report	β-Blocker prescription within 1 y	Heart failure
Antiplatelet for CAD or MI	Verified self-report	Antiplatelet prescription within 1 y	CAD or MI
β-Blocker for CAD or MI	Verified self-report	β-Blocker prescription within 1 y	CAD or MI
Statin for CAD or MI	Verified self-report	Statin prescription within 1 y	CAD or MI
Antiplatelet for CVA	Verified self-report	Antiplatelet prescription within 1 y	CVA
Controller for poorly controlled COPD	Verified self-report	ICS and LABA; LAMA and LABA; or ICS, LAMA, and LABA prescription within 1 y	COPD and systemic steroid use within 1 y

Abbreviations: ACE-I, angiotensin-converting enzyme inhibitor; ARB, angiotensin receptor blocker; CAD, coronary artery disease; COPD, chronic obstructive pulmonary disease; CVA, cerebral vascular accident; HbA_1c_, hemoglobin A_1c_; ICS, inhaled corticosteroid; LABA, long-acting β-agonist; LAMA, long-acting muscarinic antagonist; MEPS, Medical Expenditure Panel Survey; MI, myocardial infarction.

^a^ Quality indicators were adopted from previous studies.^31,32^

^b^ The MEPS collected self-reported information (via interview or self-administered survey) and verified some of the self-reported information with clinicians and pharmacies (ie, verified self-report).

### Adjustment Variables

Adjustment variables included in the regression model were age (65-74 years, 75-84 years, and ≥85 years), sex, self-reported race/ethnicity (non-Hispanic White, Hispanic, non-Hispanic Black, and other), marital status (married or not married), living arrangement (living alone or living with ≥1 person), poverty (income <100% of the federal poverty level or income ≥100% of the federal poverty level), health insurance (any private, public only, or uninsured), education attainment (less than high school, high school graduate or some college, bachelor’s degree, and more than bachelor’s degree), number of chronic conditions (0-1, 2, 3, 4, or ≥5), self-reported health (excellent or very good and good, fair, or poor), whether a participant needed assistance with activities of daily living, census region, and survey year. The number of chronic conditions was based on the list of 20 chronic conditions developed by the US Department of Health and Human Services.^33^

### Statistical Analysis

Data analysis was performed in June 2020. We first compared the characteristics of adults with dementia and those of patients without dementia with similar life expectancy. We described the quality of management of chronic diseases for each group and then examined the association between dementia status and the quality of chronic disease management (3 composite scores) using multivariable linear regression models, adjusting for the aforementioned variables. As secondary analyses, we investigated the association between dementia status and 14 individual QIs using similar multivariable linear regression models. All analyses accounted for the complex survey design of MEPS. Survey weights, sampling strata, and primary sampling unit variables were used to produce national estimates adjusted for nonresponse. All statistical analyses were conducted with Stata software, version 14.1 (StataCorp LLC). The *P* values were 2-sided, with statistical significance set at *P* < .05.

To test the sensitivity of our findings in relation to our selection of the control group, we reanalyzed the data using 2 alternative control groups: (1) any adults without dementia (regardless of estimated 5-year mortality risk) and (2) adults with cancer. We used Clinical Classification^34^ codes 11 to 45 excluding code 23 (nonmelanoma skin cancer) and code 44 (unclassified neoplasm) to identify adults with cancer in the MEPS data.^35^

## Results

### Participant Characteristics

This study included 2506 adults (mean [SD] age, 81.4 [4.7] years; 1259 [49.3%] female; 1243 [50.7%] male), of whom 1335 (53.3% [51.4%]) had a diagnosis of dementia and 1171 (47% [48.6%]) did not have a diagnosis of dementia. Adults with dementia were younger (≥85 years: 45.7% [95% CI, 42.2%-49.1%] vs 51.5% [95% CI, 47.9%-55.0%]); were more likely to have income less than 100% of the federal poverty level (17.0% [95% CI, 14.2%-19.8%] vs 13.2% [11.0%-15.4%]); were more likely to be female (65.9% [95% CI, 62.4%-69.3%] vs 31.8% [95% CI, 28.4%-35.2%]), non-Hispanic White (72.5% [95% CI, 68.8%-76.2%] vs 82.3% [95% CI, 79.7%-84.8%]), and married (33.4% [95% CI, 29.7%-37.0%] vs 43.1% [95% CI, 39.4%-46.8%]); and were more likely to live alone (42.9% [95% CI, 39.7%-46.1%] vs 36.8% [95% CI, 33.4%-40.1%]) compared with adults without dementia who had similar life expectancy (Table 2). Adults with dementia also had fewer chronic conditions and rated their general health as excellent or very good more often but were less likely to be independent in their activities of daily living compared with adults without dementia who had similar life expectancy.

**Table 2.** Characteristics of Adults With and Without Dementia^a^

Characteristic	Respondents, % (95% CI)		*P* value
	Adults with dementia (n = 1335)	Adults without dementia (n = 1171)^b^	
Age group, y			
65-74	15.0 (12.7-17.4)	8.8 (6.8-10.7)	<.001
75-84	39.3 (36.0-42.6)	39.8 (36.4-43.2)	
≥85	45.7 (42.2-49.1)	51.5 (47.9-55.0)	
Sex			
Female	65.9 (62.4-69.3)	31.8 (28.4-35.2)	<.001
Male	34.0 (30.7-37.6)	68.2 (64.8-71.6)	
Race/ethnicity			
White, non-Hispanic	72.5 (68.8-76.2)	82.3 (79.7-84.8)	<.001
Hispanic	8.5 (5.7-11.4)	5.7 (4.4-7.1)	
Black, non-Hispanic	13.1 (10.8-15.3)	8.2 (6.5-9.8)	
Other	5.9 (4.0-7.9)	3.9 (2.4-5.3)	
Census region			
Northeast	19.9 (17.1-22.7)	21.9 (17.9-25.9)	.27
Midwest	19.3 (16.3-22.4)	21.9 (18.3-25.5)	
South	38.4 (34.7-42.1)	36.8 (32.8-40.9)	
West	22.5 (19.1-25.9)	19.3 (16.2-22.4)	
Married	33.4 (29.7-37.0)	43.1 (39.4-46.8)	<.001
Living alone	42.9 (39.7-46.1)	36.8 (33.4-40.1)	.006
Educational level			
<High school	35.0 (31.3-38.6)	34.7 (31.3-38.1)	.43
High school or some college	50.0 (46.5-53.4)	47.3 (43.8-50.8)	
Bachelor’s degree	9.9 (7.8-11.9)	10.8 (8.3-13.4)	
>Bachelor’s degree	5.2 (3.2-7.3)	7.1 (5.0-9.3)	
Income			
<100% FPL	17.0 (14.2-19.8)	13.2 (11.0-15.4)	.03
≥100% FPL	83.0 (80.2-85.8)	86.8 (84.6-89.0)	
Health insurance			
Any private	42.8 (38.8-46.7)	51.9 (48.0-55.9)	<.001
Public only	57.2 (53.2-61.1)	47.6 (43.6-51.5)	
Uninsured	0.1 (0.0-0.2)	0.5 (0.0-1.0)	
Chronic conditions, No.			
0-1	28.2 (24.8-31.7)	13.8 (11.5-16.1)	<.001
2	21.5 (18.5-24.4)	15.5 (13.1-17.9)	
3	20.2 (17.5-22.8)	20.3 (17.7-22.9)	
4	13.2 (10.8-15.6)	18.9 (16.4-21.4)	
≥5	17.0 (14.6-19.4)	31.5 (28.2-34.7)	
Excellent or very good health	25.4 (22.3-28.5)	9.7 (7.5-11.9)	<.001
Needs assistance in ADLs	34.3 (30.7-37.9)	25.9 (22.7-29.1)	<.001

Abbreviations: ADLs, activities of daily living; FPL, federal poverty level.

^a^ Percentages were weighted to be nationally representative. Percentages may not add up to 100% owing to rounding.

^b^ Adults without dementia are those without a dementia diagnosis who had an estimated 5-year mortality risk of 50% or greater.

### Quality of Chronic Disease Management

In the analysis adjusted for potential confounders, adults with dementia received poorer quality of preventive care compared with adults of similar life expectancy without dementia (mean adjusted composite score, 57.7% [95% CI, 55.0% to 60.4%] vs 63.8% [95% CI, 61.3% to 66.3%]; adjusted absolute difference [aAD], −6.1 percentage points [pp] [95% CI, −9.7 to −2.5 pp]; *P* = .001) (Table 3). We found no evidence that the quality of care differed between the 2 groups in terms of diabetes care (mean adjusted composite score, 75.5% [95% CI, 70.6% to 80.5%] vs 73.8% [95% CI, 70.1% to 77.6%]; aAD, 1.7 pp [95% CI, −4.5 to 7.9 pp]; *P* = .59) and medication treatment (mean adjusted composite score, 49.5% [95% CI, 45.3% to 53.7%] vs 48.5% [95% CI, 44.9% to 52.2%]; aAD, 1.0 pp [95% CI, −5.0 to 7.0 pp]; *P* = .75). In the analysis of 14 individual QIs, the quality of chronic disease management did not differ between the 2 groups except that adults with dementia were significantly less likely to receive an influenza vaccine compared with those without dementia (adjusted percentage fulfilled, 74.5% [95% CI, 71.3% to 77.7%] vs 79.0% [95% CI, 76.0% to 81.9%]; aAD, −4.5 pp [95% CI, −8.9 to 0.0 pp]; *P* = .049) (Table 4).

**Table 3.** Comparison of the Quality of Chronic Disease Management Between Adults With and Without Dementia

Composite category	Adults with dementia (n = 1335)		Adults without dementia (n = 1171)^a^		Adjusted absolute difference^b^	
	Eligible participants, No.	Composite score, adjusted mean % (95% CI) ^b^^,^^c^	Eligible participants, No.	Composite score, adjusted mean % (95% CI)^b^^,^^c^	Coefficient, percentage points (95% CI)^d^	*P* value
Preventive care	1280	57.7 (55.0 to 60.4)	1079	63.8 (61.3 to 66.3)	−6.1 (−9.7 to −2.5)	.001
Diabetes care	314	75.5 (70.6 to 80.5)	409	73.8 (70.1 to 77.6)	1.7 (−4.5 to 7.9)	.59
Medication treatment	540	49.5 (45.3 to 53.7)	668	48.5 (44.9 to 52.2)	1.0 (−5.0 to 7.0)	.75

^a^ Adults without dementia were those without a dementia diagnosis who had an estimated 5-year mortality risk of 50% or greater.

^b^ Estimated values were weighted to be nationally representative and were adjusted for age group, sex, race/ethnicity, marital status, living alone, poverty, health insurance, education attainment, number of chronic conditions, self-reported health, assistance in activities of daily living, census region, and survey year.

^c^ Composite scores were calculated by dividing the number of processes of care delivered by the number of processes of care for which a participant was eligible in each composite category.

^d^ Positive difference indicates that adults with dementia received higher-quality care, and negative difference indicates that adults with dementia received lower-quality care.

**Table 4.** Comparison of the Quality of Chronic Disease Management Between Adults With and Without Dementia by Quality Indicator

Quality indicator	Adults with dementia (n = 1335)		Adults without dementia (n = 1171)^a^		Adjusted absolute difference^b^	
	Eligible participants, No.	Adjusted mean %^b^	Eligible participants, No.	Adjusted mean %^b^	Coefficient, percentage points (95% CI)^c^	*P* value
Preventive care						
Influenza vaccine	1243	74.5 (71.3 to 77.7)	1040	79.0 (76.0 to 81.9)	−4.5 (−8.9 to 0.0)	.049
Smoking cessation counseling	90	70.7 (59.0 to 82.3)	252	75.1 (68.8 to 81.5)	−4.4 (−17.4 to 8.5)	.50
Dental care	1270	40.9 (37.0 to 44.8)	1058	45.0 (41.3 to 48.8)	−4.1 (−9.4 to 1.2)	.13
Diabetes care						
HbA_1c_ measurement	200	86.8 (80.4 to 93.2)	249	84.1 (79.0 to 89.1)	2.7 (−6.1 to 11.6)	.54
Foot examination	301	79.5 (73.1 to 85.8)	397	77.5 (72.4 to 82.5)	2.0 (−6.6 to 10.6)	.65
Eye examination	303	67.8 (61.1 to 74.5)	403	68.1 (62.5 to 73.6)	−0.3 (−8.1 to 7.6)	.94
Medication treatment						
Anticoagulation for atrial fibrillation	125	45.5 (34.8 to 56.2)	171	47.9 (39.2 to 56.6)	−2.4 (−16.1 to 11.2)	.73
ACE-I or ARB for heart failure	95	59.2 (46.9 to 71.6)	146	52.2 (43.8 to 60.6)	7.1 (−6.7 to 20.8)	.31
β-Blocker for heart failure	95	58.0 (45.7 to 70.4)	146	57.6 (47.6 to 67.7)	0.4 (−15.4 to 16.2)	.96
Antiplatelet for CAD or MI	293	35.3 (26.9 to 43.7)	398	36.3 (30.9 to 41.6)	−0.9 (−10.6 to 8.8)	.85
β-Blocker for CAD or MI	293	65.0 (57.1 to 72.9)	398	63.8 (57.9 to 69.6)	1.2 (−9.0 to 11.5)	.81
Statin for CAD or MI	293	64.2 (56.0 to 72.4)	398	58.8 (53.1 to 64.6)	5.3 (−5.2 to 15.9)	.32
Antiplatelet for CVA	188	45.7 (36.1 to 55.3)	173	46.0 (37.1 to 54.8)	−0.3 (−14.2 to 13.7)	.97
Controller for poorly controlled COPD	20	56.7 (33.4 to 80.1)	90	33.3 (21.8 to 44.8)	23.4 (−2.9 to 49.8)	.08

Abbreviations: ACE-I, angiotensin-converting enzyme inhibitor; ARB, angiotensin receptor blocker; CAD, coronary artery disease; COPD, chronic obstructive pulmonary disease; CVA, cerebrovascular accident; HbA_1c_, hemoglobin A_1c_; MI, myocardial infarction.

^a^ Adults without dementia were those without a dementia diagnosis who had an estimated 5-year mortality risk of 50% or greater.

^b^ Estimated values were weighted to be nationally representative and were adjusted for age group, sex, race/ethnicity, marital status, living alone, poverty, health insurance, education attainment, number of chronic conditions, self-reported health, assistance in activities of daily living, census region, and survey year.

^c^ Positive difference indicates that adults with dementia received higher-quality care, and negative difference indicates that adults with dementia received lower-quality care.

### Sensitivity Analysis

Our findings were qualitatively unaffected by our selection of the control group. Adults with dementia received a lower quality of preventive care compared with any adults without dementia (aAD, −6.0 pp; 95% CI, −8.5 to −3.4 pp; *P* < .001) and adults with cancer (aAD, −6.9 pp; 95% CI, −10.4 to −3.4 pp; *P* < .001) (eTables 3 and 4 in the Supplement). Adults with dementia received a higher quality of medication treatment compared with adults with cancer (aAD, 11.0 pp; 95% CI, 4.7-17.2 pp; *P* < .001) (eTable 4 in the Supplement).

## Discussion

In this cross-sectional study of a nationally representative sample of noninstitutionalized adults 65 years or older, we found that those with dementia were less likely to receive recommended preventive care compared with adults of similar life expectancy without dementia, although the difference was small and may have been associated with the difference in receipt of influenza vaccine. We found that the quality of care for diabetes and medication treatment differed between these 2 groups. Taken together, these findings suggest that community-dwelling adults with dementia living in the US overall receive chronic disease management similar in quality to that received by those without dementia despite the potential barriers associated with cognitive impairment. Although it is critical to understand the mechanisms behind the experience of poorer quality of preventive care, our findings may provide useful information to clinicians and policymakers because the prevalence of dementia in the community is rapidly increasing in the US.^6^

We found that the composite score for preventive care was lower for adults with dementia than for adults of similar life expectancy without dementia . We also observed poorer quality of care for adults with dementia across individual QIs related to preventive care, but the difference was statistically significant only for receipt of the influenza vaccine. Several underlying mechanisms are possible. First, clinicians treating adults with dementia may have to allocate more time for salient health issues than for conversations about preventive care (eg, influenza vaccine) given that many clinicians are already under intense time pressure and that more time is required to engage in a shared decision-making discussion with the caregivers and surrogates for adults with dementia. Second, procedures and interventions that require a patient’s cooperation (eg, smoking cessation, dental examinations) may not be feasible for those with cognitive impairment and associated behaviors. Third, clinicians may underestimate the benefits of preventive care for adults with dementia given their limited life expectancy and may choose not to address preventive care.

We found no evidence that the quality of diabetes care and medication treatment differed between adults with and without dementia. The provision of these services (eg, foot examination, administration of oral medication) may be straightforward and short enough for adults with dementia, and patients may not need to be as cooperative as they need to be when certain preventive care services are provided. In addition, many chronic conditions may have been diagnosed and treated before dementia was diagnosed, and clinicians and patients often continue those treatments indefinitely unless serious adverse effects occur.

Our study builds on the results of previous studies^7,8,9,10,11,12,13,14,15,18,36,37^ that examined the association between dementia status and the quality of chronic disease management. Shah et al^7^ analyzed data from the UK and reported that adults with dementia were less likely to receive vaccinations than were those without dementia, which is consistent with our findings. Fowler et al^15^ reported that adults with dementia less frequently received guideline-recommended medications from 2 or more classes for the secondary prevention of ischemic heart disease compared with those without dementia according to cohort data from 4 US states.^15^ However, they found that the use of drugs from any particular class did not differ between the 2 groups, which is similar to our findings. More recently, Zupanic et al^13^ examined data in the Swedish National Dementia Registry and found that compared with adults without dementia, adults with dementia were less likely to be prescribed statins, antihypertensive medications, and anticoagulants during the 3 years after their first ischemic stroke associated with atrial fibrillation. Although their findings were not consistent with ours, they conducted the study using data obtained outside the US, and the inconsistency might be attributable to differences in health care systems or culture. To our knowledge, the present study is the first to assess the association of dementia status with the quality of chronic disease management for multiple conditions in a nationally representative sample of noninstitutionalized older adults in the US.

### Limitations

This study has limitations. First, although we selected QIs with careful consideration of the potential burden of care and the time horizon to benefit for adults with dementia, recommended chronic disease management specified in these QIs may not be provided on the basis of shared decision-making or patient or caregiver preferences. Such care should not be regarded as “poor” quality of care, but this cannot be assessed with a large data set such as the one we used. Second, because of limited availability of data, we could not include other preventive care services that were relevant to the study population, such as pneumococcal vaccination and routine determination of body weight. Further research is warranted to assess whether our findings are generalizable to other preventive care services. Third, a diagnosis of dementia in our study depended on self-report or proxy report and, therefore, may be susceptible to misclassification. However, findings from a previous study suggest that medical conditions reported by MEPS respondents are generally accurate.^38^ If a misclassification exists, the direction of bias would be toward the null (ie, underestimation of the differences) given that dementia diagnoses are often underreported.^39^ Our analysis was not intended to uncover the mechanism of the difference (or lack of difference) in the quality of chronic disease management between adults with and without dementia. Future research is needed to better understand the association between dementia status and chronic disease management.

## Conclusions

In this cross-sectional study, using a nationally representative sample of noninstitutionalized adults 65 years or older in the US, we found that adults with dementia were less likely than adults without dementia who had similar life expectancy to receive preventive care (particularly influenza vaccines), although diabetes care and medication treatment did not differ between the 2 groups. In general, adults with dementia appeared to receive recommended chronic disease management as frequently as those without dementia. Our study highlights the need to better understand the reasons that adults with dementia may be unlikely to receive preventive care to further improve the quality of care in this rapidly increasing population.

## Notes

Supplement. **eTable 1.** Comparison of 5-Year Mortality Index Construct Between NHIS and MEPS **eTable 2.** Experts’ Opinion on Time Horizon to Benefit and Potential Burden of Care from Assessing Care of Vulnerable Elders-3 (ACOVE-3) **eTable 3.** Comparison of Chronic Disease Management Between Adults with Dementia and Those Without Dementia (With Any Estimated 5-Year Mortality Risk) **eTable 4.** Comparison of Chronic Disease Management Between Adults With Dementia and Those With Cancer **eFigure.** Flowchart of the Study Population eReference.
