# Supplementary material for: Comparison of the Quality of Chronic Disease Management Between Adults With and Without Dementia
Source: JAMA Netw Open. 2021 May 13;4(5):e219622. doi: 10.1001/jamanetworkopen.2021.9622 (PMC8120327; doi:10.1001/jamanetworkopen.2021.9622)
Supplement: Supplement. — eTable 1. Comparison of 5-Year Mortality Index Construct Between NHIS and MEPS eTable 2. Experts’ Opinion on Time Horizon to Benefit and Potential Burden of Care from Assessing Care of Vulnerable Elders-3 (ACOVE-3) eTable 3. Comparison of Chronic Disease Management Between Adults with Dementia and Those Without Dementia (With Any Estimated 5-Year Mortality Risk) eTable 4. Comparison of Chronic Disease Management Between Adults With Dementia and Those With Cancer eFigure. Flowchart of the Study Population eReference. [file jamanetwopen-e219622-s001.pdf]

Supplementary Online Content

Gotanda H, Nuckols T, Mori K, Tsugawa Y. Comparison of the quality of chronic disease management between adults with and without dementia. *JAMA Netw Open*. 2021;4(5):e219622. doi:10.1001/jamanetworkopen.2021.9622

eTable 1. Comparison of 5-Year Mortality Index Construct Between NHIS and MEPS
eTable 2. Experts' Opinion on Time Horizon to Benefit and Potential Burden of Care from Assessing Care of Vulnerable Elders-3 (ACOVE-3)
eTable 3. Comparison of Chronic Disease Management Between Adults with Dementia and Those Without Dementia (With Any Estimated 5-Year Mortality Risk)
eTable 4. Comparison of Chronic Disease Management Between Adults With Dementia and Those With Cancer
eFigure. Flowchart of the Study Population
eReference.

This supplementary material has been provided by the authors to give readers additional information about their work.

© 2021 Gotanda H et al. *JAMA Network Open*.

eTable 1. Comparison of 5-year mortality index construct between NHIS and MEPS

	NHIS (original)	pts		MEPS	pts
1	Age			Age	
	65-69	0		65-69	0
	70-74	1		70-74	1
	75-79	3		75-79	3
	80-84	5		80-84	5
	85+	7		85+	7
2	Sex			Sex	
		2		Female	2
3	BMI			BMI	
	<25	2		<25	2
4	Would you say your health in general is:			In general, compared to other people of your age, would you say that your health is:	
	Excellent/Very good	0		Excellent/Very good	0
	Good	1		Good	1
	Fair/Poor	2		Fair/Poor	2
5	Have you ever been told by a doctor or health professional that you had:			Diagnoses based on Clinical Classification Software (CCS) codes in MEPS Medical Condition files:	
	Emphysema/chronic bronchitis	2		Emphysema/chronic bronchitis	2
	A cancer (excluding non-melanoma skin cancer)	2		A cancer (excluding non-melanoma skin cancer)	2
	Diabetes (include borderline diabetes)	2		Diabetes (include borderline diabetes)	2
6	Because of a physical mental, or emotional problems, do you need the help of other persons in handling routine needs such as everyday household chores, doing necessary business, shopping, or getting around for other purposes?			Do you receive help or supervision using the telephone, paying bills, taking medications, preparing light meals, doing laundry, or going shopping (because of an impairment or a physical or mental health problem)?	
	No	0		No	0
	Yes	2		Yes	2
7	By yourself, and without using any special equipment, how difficult is it for you to walk a quarter of a mile (about 3 city blocks)?			How much difficulty do you have walking about 3 city blocks or about a quarter of a mile?	
	Not at all difficult	0		No difficulty	0
	A little difficult to very difficult	3		Some difficulty/A lot of difficulty	3
	Can't do at all/do not do	3		Completely unable to do it	3
8	Which best describes your cigarette use?			Do you currently smoke?	
	Never smoked (<100 cigarettes in your entire life)	0		No	0
	Former smoker	1			
	Current smoker (smoke some days or every day)	3		Yes	3
9	During the past 12 months, how many times were you hospitalized overnight?			Overnight hospitalization based on the MEPS Hospital Inpatient Stays files:	
	None	0		None	0
	Once	1		Once	1
	Twice or more	3		Twice or more	3

Notes: This table was modified from Gotanda H, Ganz DA, Wenger NS. Association Between Estimated Mortality Risk and Measured Quality of Care in Older Adults. J Am Geriatr Soc. 2018;66(9):1838-44. Abbreviations: BMI, body mass index. MEPS, Medical Expenditure Panel Survey; NHIS, National Health Interview Survey.

© 2021 Gotanda H et al. *JAMA Network Open*.

eTable 2. Experts' opinion on time horizon to benefit and potential burden of care from Assessing Care of Vulnerable Elders-3 (ACOVE-3)

Quality indicators	Care to be provided to fulfill an indicator	Eligibility for an indicator	Time horizon to benefit	Potential burden of care
**Preventive care**				
Influenza vaccine	Influenza vaccine within 1y	Age ≥50 y	Short	Low
Smoking cessation counseling	Smoking cessation counseling within1y	Smokers	Intermediate	Low
Dental care	Dental visit within 1y	All	Short	Low
**Diabetes care**				
HbA1c measurement	HbA1c measurement at least twice yearly	Diabetes	Long*	Low
Foot examination	Foot examination within 1y	Diabetes	Short	Low
Eye examination	Eye examination within 1y	Diabetes	Long*	Low
**Medication treatment**				
Anticoagulation for atrial fibrillation	Anticoagulation prescription within 1y	Atrial fibrillation	Short	Moderate
ACE-I/ARB for heart failure	ACE-I/ARB prescription within 1y	Heart failure	Intermediate	Low
β-blocker for heart failure	β-blocker prescription within 1y	Heart failure	Intermediate	Low
Antiplatelet for CAD/MI	Antiplatelet prescription within 1y	CAD or MI	Intermediate	Low
Beta-blocker for CAD/MI	Beta-blocker prescription within 1y	CAD or MI	Intermediate	Low
Statin for CAD/MI	Statin prescription within 1y	CAD or MI	Intermediate	Low
Antiplatelet for CVA	Antiplatelet prescription within 1y	CVA	Short	Low
Controller for poorly controlled COPD	ICS+LABA or LAMA+LABA or ICS+LAMA+LABA prescription within 1y	COPD and systemic steroid use within 1y	Short	Low

Notes: This table was modified from Wenger NS, Solomon DH, Amin A, Besdine RK, Blazer DG, Cohen H, et al. Application of Assessing Care of Vulnerable Elders-3 Quality Indicators to Patients with Advanced Dementia and Poor Prognosis. J Am Geriatr Soc. 2007;55 Suppl 2:S457-63. *We included these measures in diabetes care composite based on a prior study,[1] even though the experts in the ACOVE-3 project determined that these measures have a "long" time horizon. Abbreviations: HbA1c, hemoglobin A1c; ACE-I, angiotensin-converting enzyme inhibitor; ARB, angiotensin receptor blocker; CAD coronary artery disease; COPD, chronic obstructive pulmonary disease; CVA, cerebral vascular accident; ICS, inhaled corticosteroid; LABA, long-acting β agonist; LAMA, long-acting muscarinic antagonist; MI, myocardial infarction.

© 2021 Gotanda H et al. *JAMA Network Open*.

eTable 3. Comparison of chronic disease management between adults with dementia and those without dementia (with any estimated 5-year mortality risk)

**Composite category**	**Adults with dementia (n=1,335)**		**Adults without dementia (n=27,128)**		**Adjusted absolute difference**	
	Eligible participants, No.	Composite score, adjusted mean %	Eligible participants, No.	Composite score, adjusted mean %	Coefficient, percentage point (95% CI)	*P* value
Preventive care	1280	61.0	26742	66.9	-6.0 (-8.5 to -3.4)	<.001
Diabetes care	314	74.1	5434	75.1	-1.1 (-5.8 to 3.6)	.66
Medication treatment	540	47.8	7020	47.9	-0.0 (-4.4 to 4.3)	.99

Notes: Adults without dementia are those without dementia diagnosis regardless of estimated 5-year mortality risk (see main text for more details). See the notes for Table 3 in the main text for more details.

eTable 4. Comparison of chronic disease management between adults with dementia and those with cancer

**Composite category**	**Adults with dementia (n=1,335)**		**Adults without dementia (n=27,128)**		**Adjusted absolute difference**	
	Eligible participants, No.	Composite score, adjusted mean %	Eligible participants, No.	Composite score, adjusted mean %	Coefficient, percentage point (95% CI)	*P* value
Preventive care	1157	63.8	2676	70.7	-6.9 (-10.4 to -3.4)	<.001
Diabetes care	289	75.3	582	77.8	-2.5 (-8.8 to 3.7)	.43
Medication treatment	477	55.4	907	44.4	11.0 (4.7 to 17.2)	<.001

Notes: Adults with cancer are those without dementia diagnosis who have cancer (see main text for more details). See the notes for Table 3 in the main text for more details.

eFigure. Flowchart of the study population

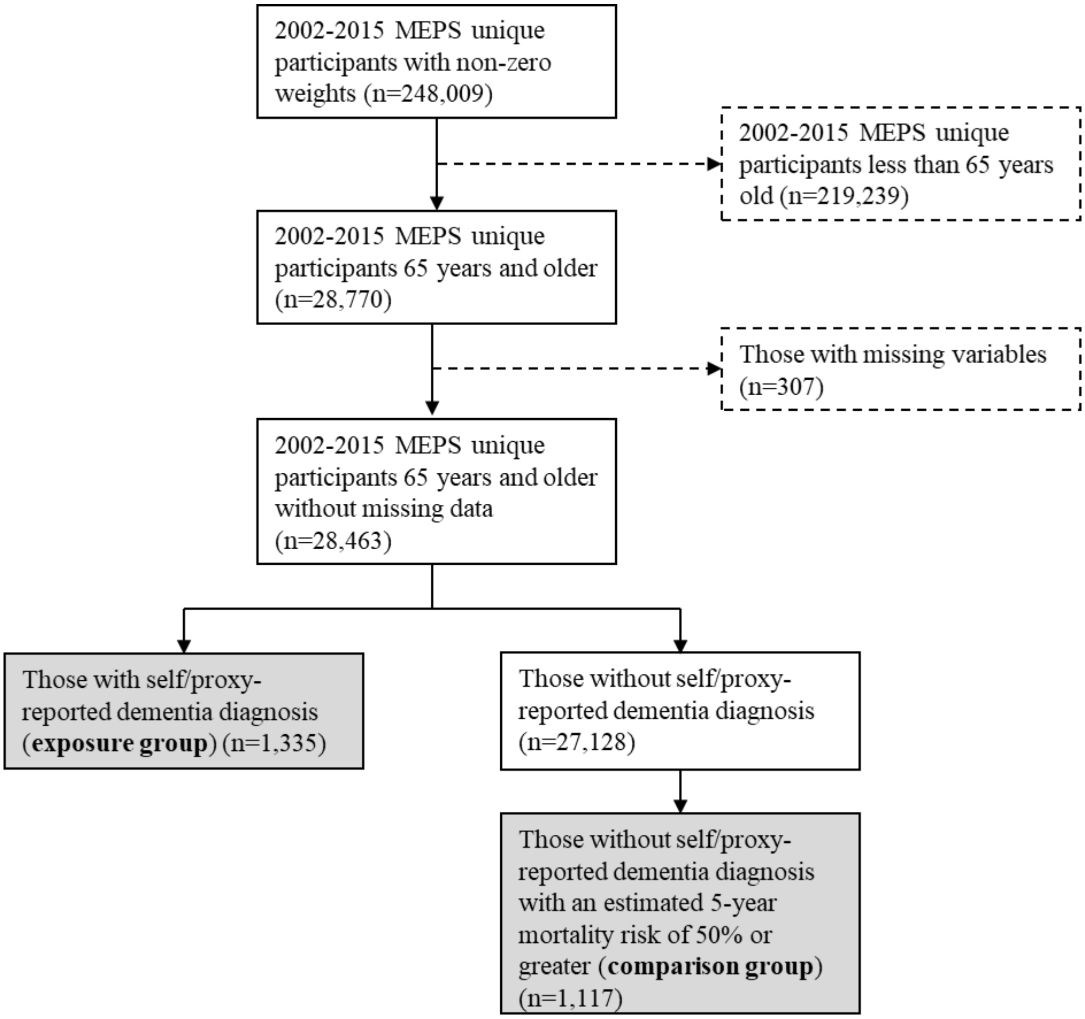

© 2021 Gotanda H et al. *JAMA Network Open*.

Supplemental Reference

1. Thorpe CT, Thorpe JM, Kind AJH, Bartels CM, Everett CM, Smith MA. Receipt of monitoring of diabetes mellitus in older adults with comorbid dementia. J Am Geriatr Soc. 2012;60(4):644-651.

© 2021 Gotanda H et al. *JAMA Network Open*.
